# Supplementary material for: Altered G1 signaling order and commitment point in cells proliferating without CDK4/6 activity
Source: Nat Commun. 2020 Oct 20;11:5305. doi: 10.1038/s41467-020-18966-9 (PMC7576148; doi:10.1038/s41467-020-18966-9)
Supplement: Supplementary file 1 — Supplementary Information [file 41467_2020_18966_MOESM1_ESM.pdf]

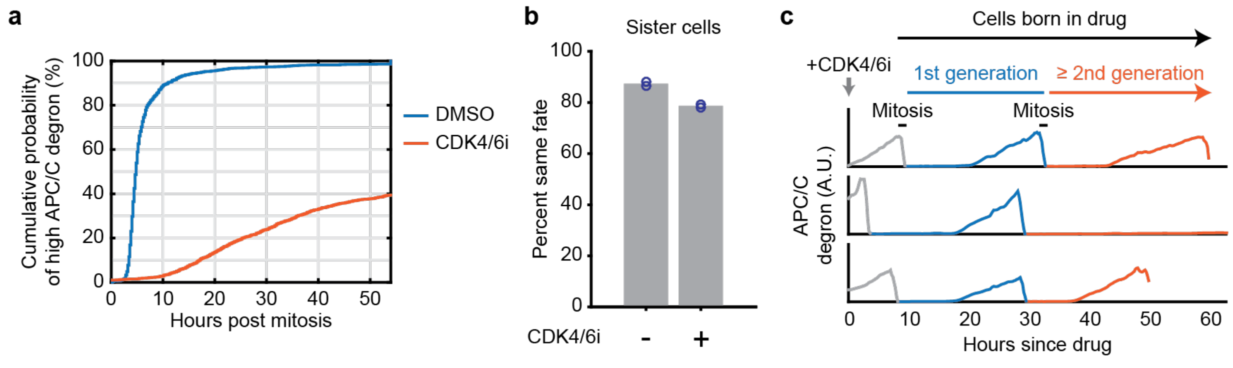

Supplementary Figure 1. Related to Figure 1

a, CDF of asynchronously cycling cells inactivating APC/C^{CDH1} after mitosis. n=1643 and 4178 cells for DMSO and CDK4/6i, respectively.1 of n=2 biological replicates. **b,** Percent sister cells with the same fate as assayed by APC/C inactivation. Mean of n=2 biological replicates. **c,** Example live-cell traces demonstrating separation of first and subsequent generations in MCF-10A cells. Example cells were born into CDK4/6 inhibitor and drug was refreshed every 24hrs.

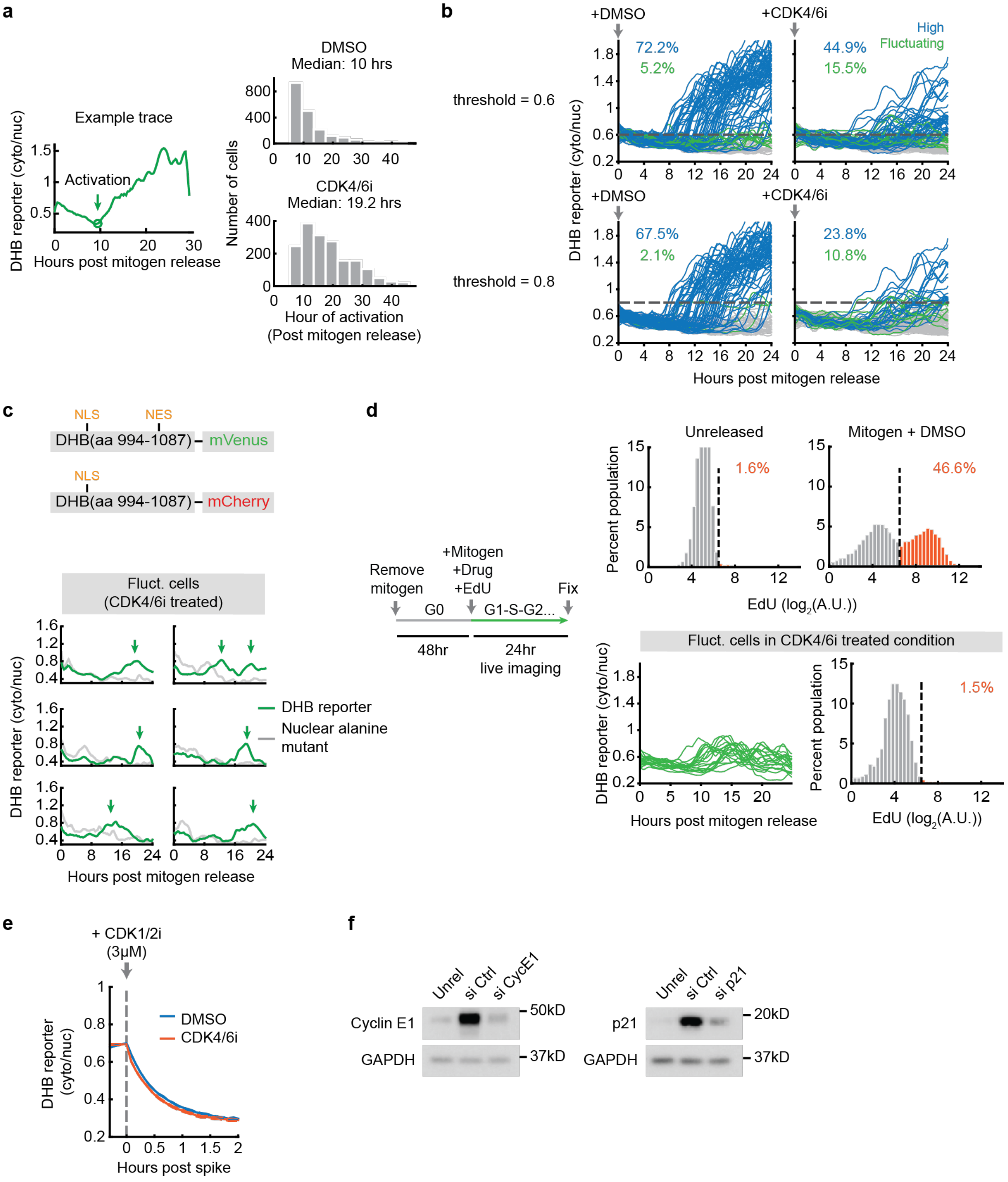

Supplementary Figure 2. Related to Figure 2. Delayed and fluctuating cyclin E/A-CDK activity in CDK4/6 inhibitor-treated cells

a, Left: Example trace of cyclin E/A-CDK activity with circle denoting point of activation. Right: Distributions of activation time in cells coming out of mitogen release. DMSO: 2189 cells; CDK4/6i: 2508 cells. **b,** Data from Figure 2b reanalyzed using thresholds of 0.6 and 0.8 (horizontal dashed line) instead of 0.7. 100 cells plotted; 2782 and 8548 cells total for DMSO and CDK4/6 inhibitor; 1 of n=2 biological replicates. **c,** Top: Co-transfected constructs. Bottom: Example traces

of cells expressing both DHB reporter (green) and nuclear DHB-alanine-mutant reporter (gray). Green arrows denote fluctuations not due to analysis noise. **d,** Left: cells were mitogen-released with EdU to assay for S-phase entry. Middle two histograms: negative and positive controls for determining EdU threshold. Right: EdU incorporation in cyclin E/A-CDK fluctuating cells (<0.7 at last time point, exceeded 0.7 at some point between 8 and 24hrs after mitogen release). 20 fluctuating cells plotted for the traces, 474 fluctuating cells total. **e,** Cyclin E/A-CDK activity of DMSO and CDK4/6i-treated cells that were treated with an additional 3μM of CDK1/2i. Data presented as median values ± 95% confidence interval based on 630 and 296 cells for DMSO and CDK4/6 inhibitor conditions. Cells from 1 of n=2 biological replicates. **f,** Western blot validation for cyclin E1 and p21 knockdown. si Ctrl and si p21 samples were collected 12hrs after mitogen release.

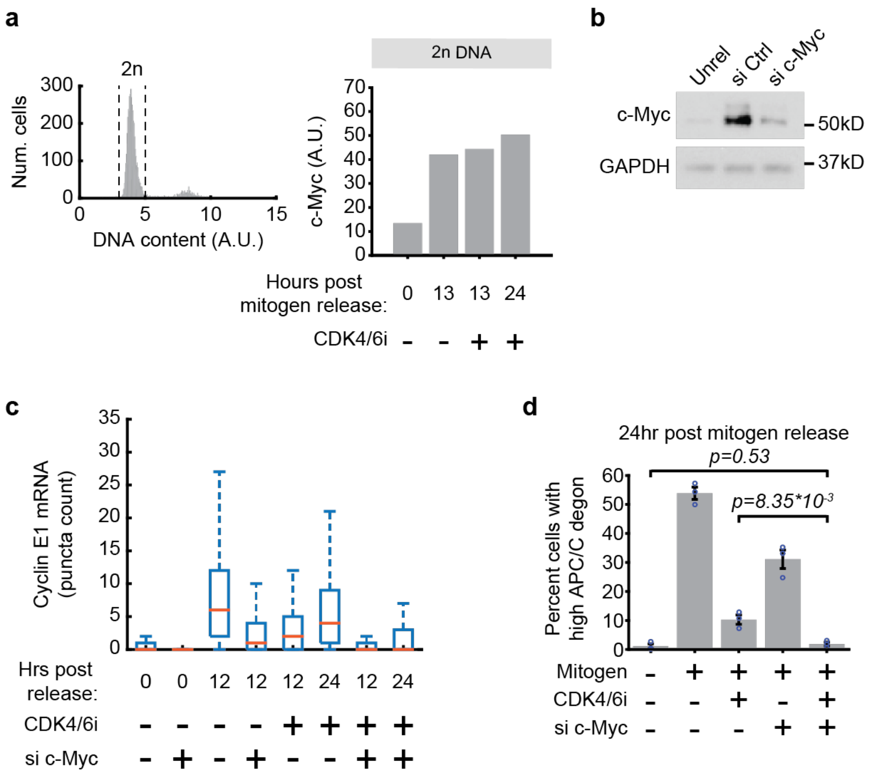

Supplementary Figure 3. Related to Figure 2. c-Myc is necessary for APC/C^{CDH1} inactivation in CDK4/6 inhibitor-treated cells

a, Left: 2n DNA gating explanation. Right: median c-Myc protein level as measured by immunofluorescence. From left to right in bar graph: n=26364, 21925, 22773, 22244 cells. **b,** Western blot validation for c-Myc knockdown. si Ctrl and si c-Myc samples were collected 12hrs after mitogen release. **c,** Cyclin E1 mRNA puncta count in cells mitogen released with and without CDK4/6 inhibitor and si c-Myc (from left to right, n=5992, 8133, 10381, 9584, 8628, 6132, 9535, and 4681 cells). Box center indicates median, box edges denote quartiles, and whiskers extend to 1.5 times the interquartile range away from the box edges. **d,** Cells with and without CDK4/6 inhibitor and si c-Myc were fixed 24hrs after mitogen release. Mean ± SEM from 3 biological replicates; p-values calculated using two-sided, two-sample t-tests.

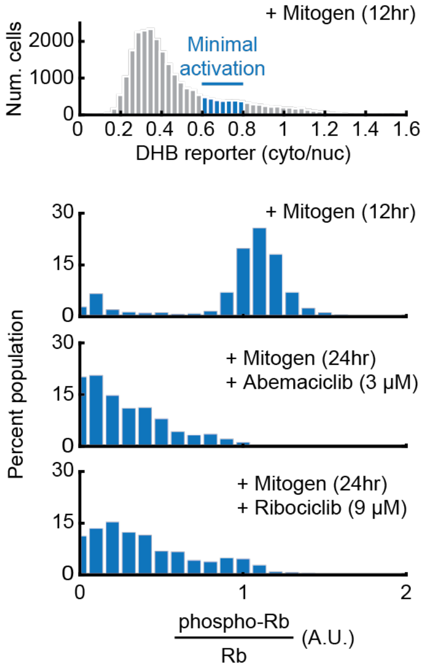

Supplementary Figure 4. Related to Figure 3. Cyclin E/A-CDK activation without phosphorylated Rb in cells treated with different CDK4/6 inhibitors

Cells expressing the cyclin E/A-CDK activity reporter and APC/C degron reporter were mitogen-released, fixed after 12hrs (DMSO) and 24hrs (Abemaciclib at 3μM and Ribociclib at 9μM), and stained for Rb and phospho-Rb(S807/S811). G1 cells with recently activated cyclin E/A-CDK activity were analyzed. n= 25622, 29553, and 28693 cells for DMSO, Abemaciclib, and Ribociclib conditions, respectively.

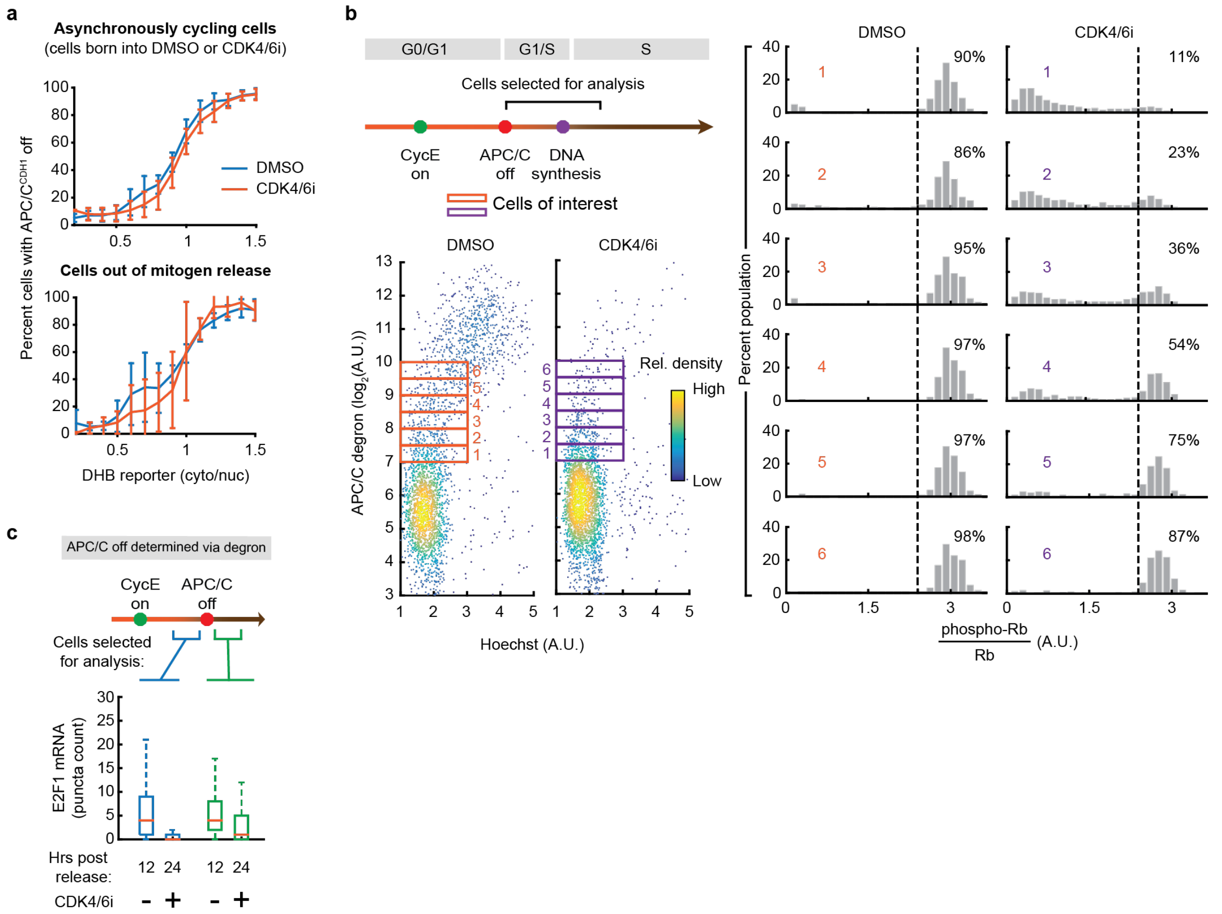

Supplementary Figure 5. Related to Figure 4. Rb phosphorylation and E2F activation occurs after APC/C^{CDH1} inactivation in CDK4/6-inhibited cells

a, The percentages of cells with APC/C^{CDH1} inactivated were determined for each bin of the cyclin E/A-CDK activity. Top: Cells born into DMSO or CDK4/6 inhibitor. Mean ± SD of 3 biological replicates. Bottom: Cells mitogen-released with DMSO or CDK4/6 inhibitor. Mean ± SD of 4 biological replicates. **b,** Asynchronously cycling cells born into DMSO or 1µM CDK4/6 inhibitor and have recently inactivated APC/C^{CDH1} were analyzed for phospho-Rb(S807/S811) signal (3000 cells plotted for scatter plots. >180 cells per histogram for DMSO, >350 cells per histogram for CDK4/6 inhibitor; 1 of n=2 biological replicates). **c,** E2F1 mRNA in cells released with or without CDK4/6 inhibitor. High cyclin E/A-CDK activity defined as ratio of signal>0.7. APC/C^{CDH1} inactivation determined via the degron-based activity reporter. From left to right, n=8747, 4063, 1961, and 189 cells). Box center indicates median, box edges denote quartiles, and whiskers extend to 1.5 times the interquartile range away from the box edges. 1 of n=2 biological replicates.

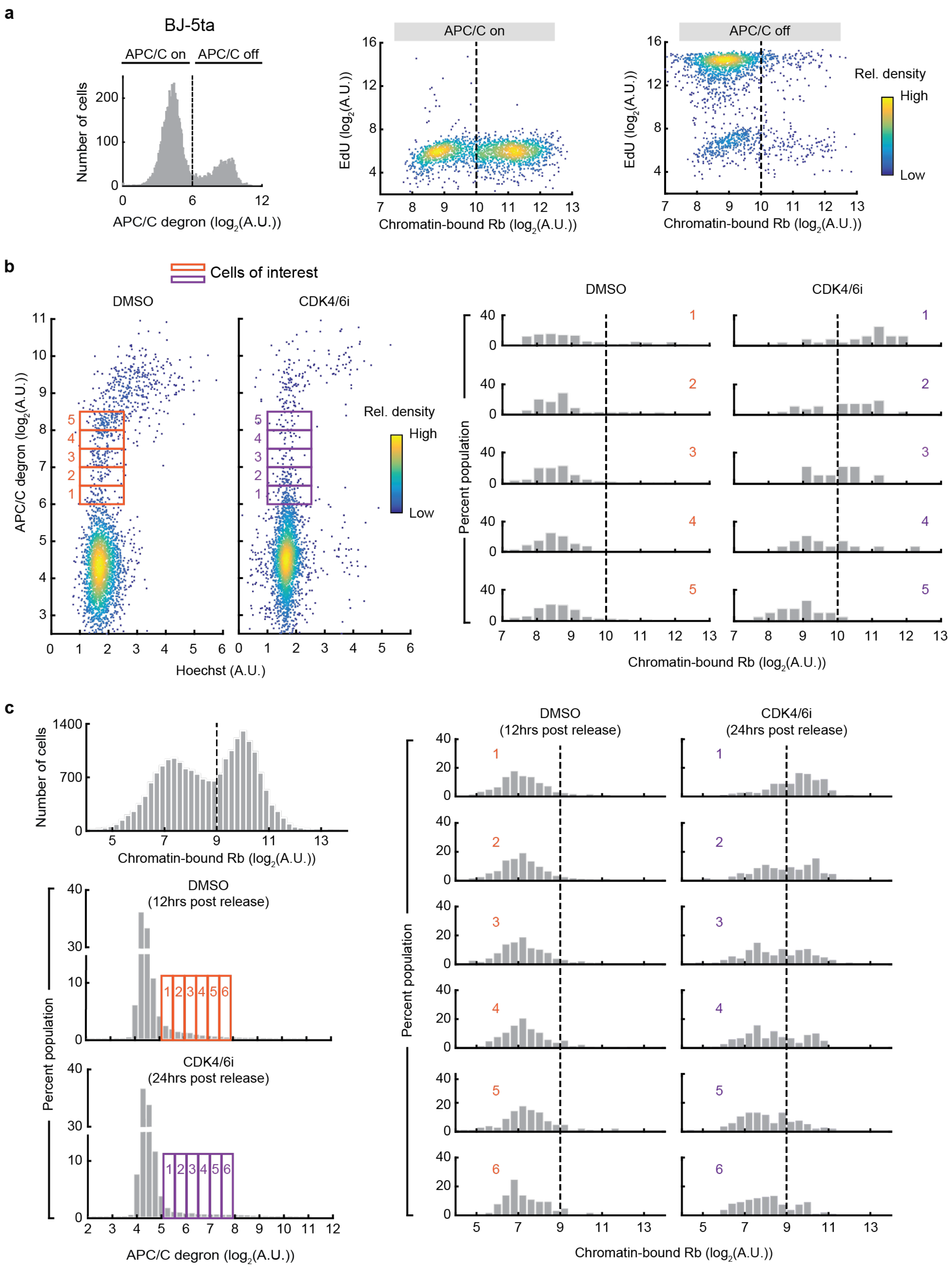

Supplementary Figure 6. Related to Figure 4. Validation and quantification of chromatin-bound Rb in CDK4/6-inhibited cells.

a, BJ-5ta cells were treated with CDK4/6 inhibitor for 96hrs and 100µM EdU for the final 5min, then permeabilized/pre-extracted, fixed, and separated into APC/C on and APC/C off. Cells were then assayed for EdU and Rb signals (2000 cells plotted). Drugs were refreshed every 24hrs. **b,** Cells from **a** that recently inactivated APC/C^{CDH1} were analyzed for pre-extracted Rb signal. >100 cells per histogram for DMSO, >5 cells per histogram for CDK4/6 inhibitor. **c,** MCF-10A cells were mitogen-released for 12hrs (with DMSO) or 24hrs (with CDK4/6 inhibitor), pre-extracted, and fixed. Cells that have recently inactivated APC/C^{CDH1} were analyzed for pre-extracted Rb signal. >60 cells per histogram for DMSO, >110 cells per histogram for CDK4/6 inhibitor. 1 of n=2 biological replicates.

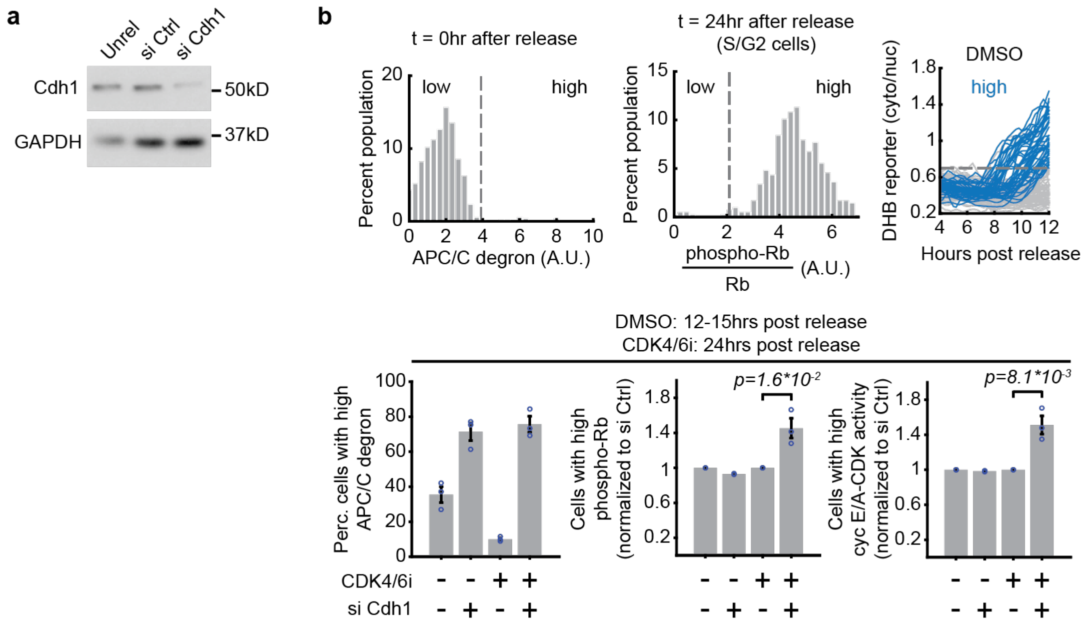

Supplementary Figure 7. Related to Figure 4. Accelerated APC/C^{CDH1} inactivation results in increased Rb inactivation and cyclin E-CDK2 activation

a, Western blot validation for Cdh1 knockdown. si Ctrl and si Cdh1 samples were collected 12hrs after mitogen release. **b,** Top: Cells were grouped into low or high APC/C degron, phospho-Rb signal, or cyclin E/A-CDK activity. Bottom: Percentages of mitogen released MCF-10A cells with high APC/C degron, phospho-Rb signal, or cyclin E/A-CDK activity after knockdown of Cdh1. With the exception of APC/C degron, percentages were normalized to si Ctrl. Mean ± SEM from 3 biological replicates; p-values calculated using two-sided, two-sample t-tests.

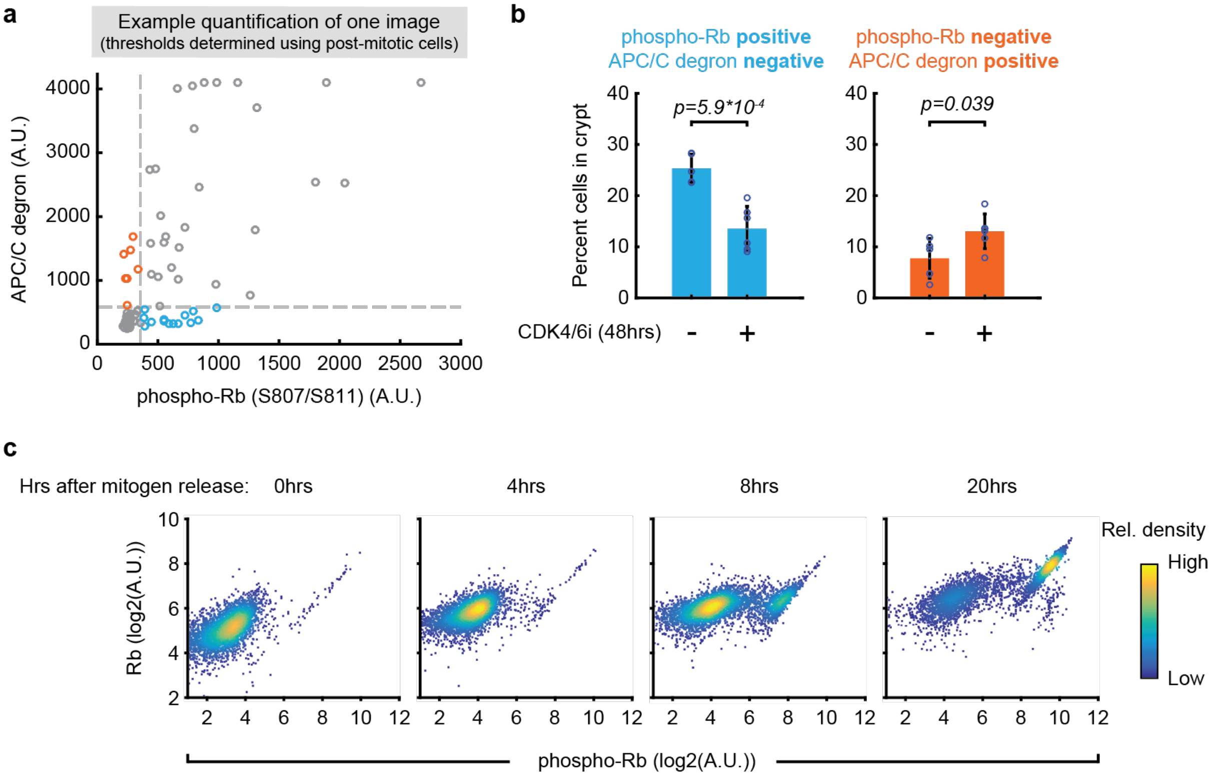

Supplementary Figure 8. Related to Figure 5. Quantification of small intestinal crypt cells
a, Example scatter plot demonstrating the quantification process. Thresholds determined unbiasedly using post-mitotic cells, which have low phospho-Rb and APC/C degron signals. **b,** Quantification of percent cells with high phospho-Rb, low APC/C degron signal and vice versa. n=5 mice for control and n=6 mice for CDK4/6 inhibitor. Mean ± SD. At least 100 intestinal crypt cells are quantified per mouse. p-values calculated using two-sided, two-sample t-tests. **c,** Rb and phospho-Rb (S807/S811) measured in mitogen released MCF-10A cells. 5000 cells plotted per condition. 1 of n=3 biological replicates.

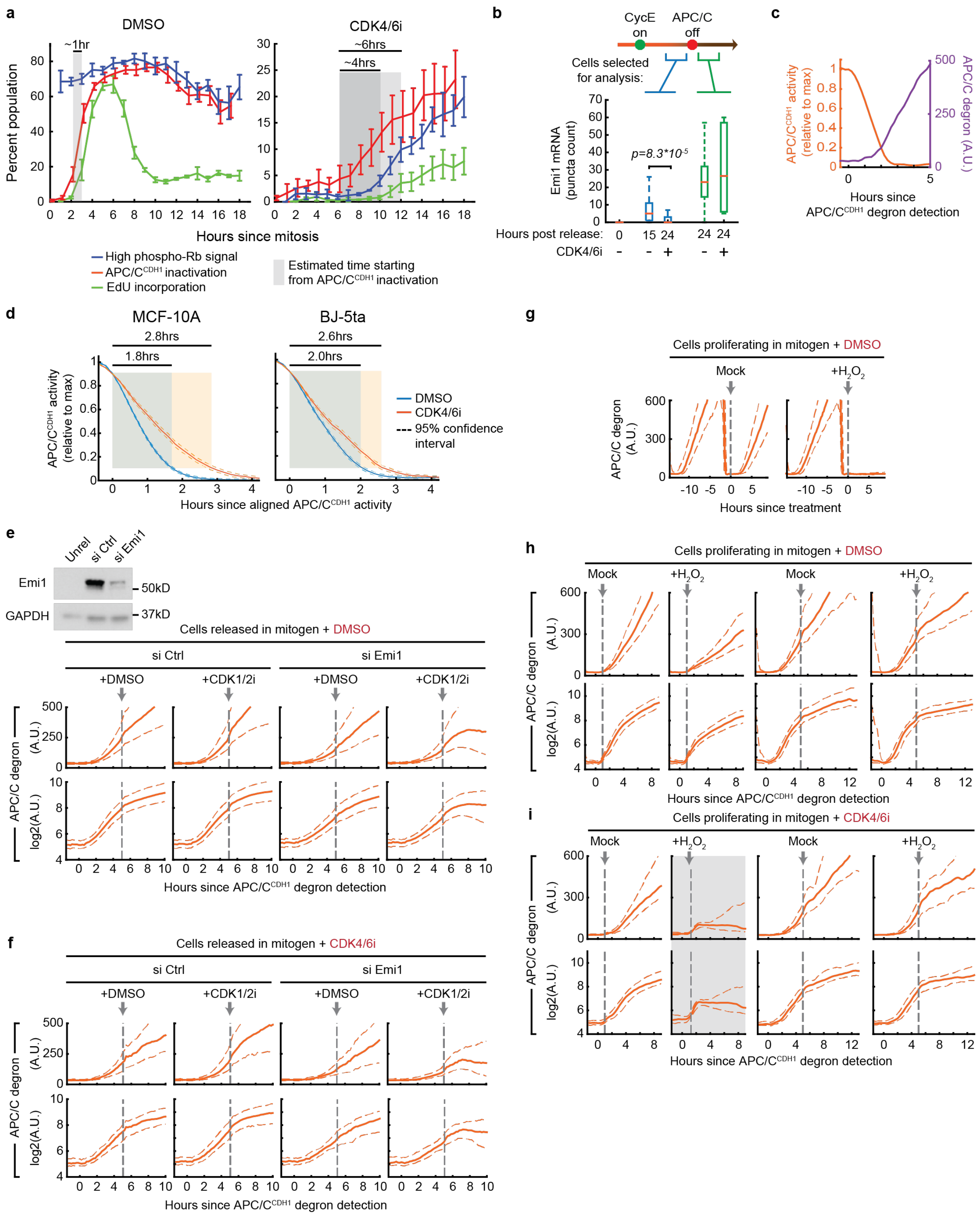

Supplementary Figure 9. Related to Figure 6. Emi1 confers irreversibility to APC/C^{CDH1} inactivation in the canonical and alternate pathway

a, Cells born into DMSO or CDK4/6 inhibitor were aligned at the time of birth. For each hour after mitosis, the percent cells with high phospho-Rb(S807/S811) signal, high APC/C degron, and high EdU signal (after 15min of 10µM EdU treatment) were calculated. Mean ± SEM from 3 biological

replicates for EdU and APC/C degron, 2 biological replicates for phospho-Rb. **b,** EMI1 mRNA in cells that were mitogen-released with or without 1μM CDK4/6 inhibitor. High cyclin E/A activity defined as reporter ratio>0.7. p-values calculated using two-sided, two-sample t-tests (n=44 and 95 cells for DMSO and CDK4/6 inhibitor conditions, respectively). Box center indicates median, box edges denote quartiles, and whiskers extend to 1.5 times the interquartile range away from the box edges. 1 of n=3 biological replicates. **c,** Example of APC/C degron conversion to APC/C^{CDH1} activity. **d,** Calculated APC/C^{CDH1} inactivation kinetics in cells born into DMSO or CDK4/6 inhibitor. Shaded boxes denote 90% to 10% activity. Mean with 95% confidence interval (MCF-10A: n=3357 cells in DMSO and 601 cells in CDK4/6 inhibitor; BJ-5ta: n=1258 cells in DMSO and 322 cells in CDK4/6 inhibitor). **e-f,** Cells that were mitogen released into DMSO or CDK4/6 inhibitor and have inactivated APC/C^{CDH1} were treated with 3μM CDK1/2i and si Emi1 (western blot knockdown validation in **e**, siRNA samples collected 24hrs after mitogen release). Cells pooled from 4 biological replicates. Dashed lines denote 25th and 75th percentile. >100 cells per condition. Bottom row panels plotted in $\log_2$ scale for better visualization. **g-i,** Cells born into DMSO or CDK4/6 inhibitor and have inactivated APC/C^{CDH1} (with the exception of **g**, where it is just cells born into DMSO or CDK4/6 inhibitor) were treated with 200μM H_2O_2. Dashed lines denote 25th and 75th percentile; bottom row panels plotted in $\log_2$ scale for better visualization. APC/C degron detected via signal over background. >25 cells per condition. In **h-i**, to enrich for Rb inactivated cells in the 5hrs post APC/C^{CDH1} degron detection conditions, only cells with cyclin E/A-CDK>0.8 right before treatment were analyzed (threshold determined from Figure 3e). Gray box denotes conditions where cells re-activated APC/C^{CDH1}.

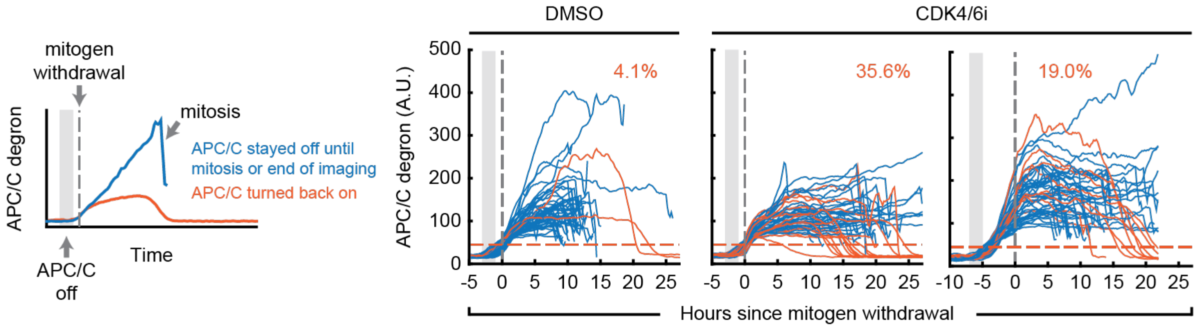

Supplementary Figure 10. Related to Figure 6. Restriction point analysis in cells with minimal CDK4/6 activity

Left: Mitogen released cells were aligned based on timing of APC/C^{CDH1} inactivation relative to mitogen withdrawal. Cell fate was then classified depending on whether APC/C^{CDH1} remained off (blue) or turned back on before mitosis (orange). Right: APC/C degron in cells mitogen released with or without CDK4/6i. Gray boxes indicate the time cells turned off APC/C^{CDH1}, and vertical dashed lines indicate time of mitogen withdrawal. Unless cells have completed mitosis, APC/C degron that fall below the orange horizontal dashed line are classified as APC/C reactivated. 50 cells plotted. From left to right: n = 542, 174, 189 cells.

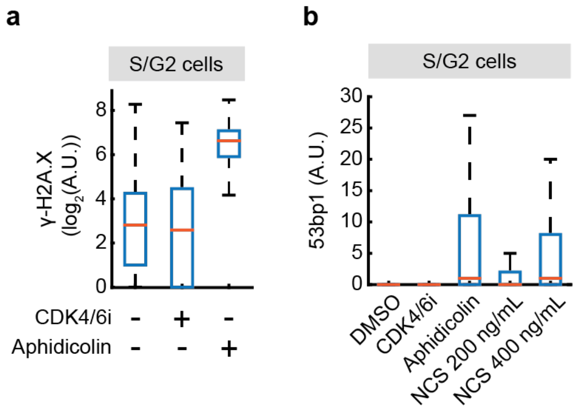

Supplementary Figure 11. Related to Figure 6. Cells proliferating without CDK4/6 activity have a viable S phase

a, Box plots of phospho-γH2A.X(S139) puncta count in S/G2 cells. Cells were treated with 1μM CDK4/6 inhibitor or 1μM aphidicolin (positive control) for 48hrs, drugs were refreshed at 24hrs (10421, 2319, 3850 cells for DMSO, CDK4/6 inhibitor, and aphidicolin, respectively). Box center indicates median, box edges denote quartiles, and whiskers extend to 1.5 times the interquartile range away from the box edges. **b,** 53BP1 puncta count in S/G2 cells. Cells were treated with 1μM CDK4/6 inhibitor for 48hrs. Positive controls: 1μM aphidicolin (48hrs) and NCS (15min pulse 24hrs before fixation). Drugs were refreshed at 24hrs and only S/G2 cells were quantified (10223, 3406, 1931, 9326, 6619 cells for DMSO, CDK4/6 inhibitor, aphidicolin, NCS low, and NCS high, respectively). Box center indicates median, box edges denote quartiles, and whiskers extend to 1.5 times the interquartile range away from the box edges.
