# Supplementary material for: Altered G1 signaling order and commitment point in cells proliferating without CDK4/6 activity
Source: Nat Commun. 2020 Oct 20;11:5305. doi: 10.1038/s41467-020-18966-9 (PMC7576148; doi:10.1038/s41467-020-18966-9)
Supplement: Supplementary file 3 — Reporting Summary [file 41467_2020_18966_MOESM3_ESM.pdf]

nature research

Corresponding author(s): Tobias Meyer

Last updated by author(s): 8/23/2020

# Reporting Summary

Nature Research wishes to improve the reproducibility of the work that we publish. This form provides structure for consistency and transparency in reporting. For further information on Nature Research policies, see our Editorial Policies and the Editorial Policy Checklist.

## Statistics

For all statistical analyses, confirm that the following items are present in the figure legend, table legend, main text, or Methods section.

| n/a | Confirmed | |
| --- | --- | --- |
| ☐ | ☒ | The exact sample size ($n$) for each experimental group/condition, given as a discrete number and unit of measurement |
| ☐ | ☒ | A statement on whether measurements were taken from distinct samples or whether the same sample was measured repeatedly |
| ☐ | ☒ | The statistical test(s) used AND whether they are one- or two-sided<br>*Only common tests should be described solely by name; describe more complex techniques in the Methods section.* |
| ☒ | ☐ | A description of all covariates tested |
| ☒ | ☐ | A description of any assumptions or corrections, such as tests of normality and adjustment for multiple comparisons |
| ☐ | ☒ | A full description of the statistical parameters including central tendency (e.g. means) or other basic estimates (e.g. regression coefficient) AND variation (e.g. standard deviation) or associated estimates of uncertainty (e.g. confidence intervals) |
| ☐ | ☒ | For null hypothesis testing, the test statistic (e.g. $F$, $t$, $r$) with confidence intervals, effect sizes, degrees of freedom and $P$ value noted<br>*Give P values as exact values whenever suitable.* |
| ☒ | ☐ | For Bayesian analysis, information on the choice of priors and Markov chain Monte Carlo settings |
| ☒ | ☐ | For hierarchical and complex designs, identification of the appropriate level for tests and full reporting of outcomes |
| ☒ | ☐ | Estimates of effect sizes (e.g. Cohen's $d$, Pearson's $r$), indicating how they were calculated |

*Our web collection on statistics for biologists contains articles on many of the points above.*

## Software and code

Policy information about availability of computer code

| | |
| --- | --- |
| Data collection | MetaExpress version 6.1, a software provided by Molecular Devices, was used for acquisition of microscopy images on ImageXpress Micro. For tissue slices, a FV1000/IX83 confocal microscope (Olympus) was used. |
| Data analysis | Matlab code based on the ones deposited in https://github.com/scappell/Cell_tracking was used for image and data analysis. For tissue slices, images were segmented in ImageJ. |

For manuscripts utilizing custom algorithms or software that are central to the research but not yet described in published literature, software must be made available to editors and reviewers. We strongly encourage code deposition in a community repository (e.g. GitHub). See the Nature Research guidelines for submitting code & software for further information.

## Data

Policy information about availability of data

All manuscripts must include a data availability statement. This statement should provide the following information, where applicable:

- Accession codes, unique identifiers, or web links for publicly available datasets
- A list of figures that have associated raw data
- A description of any restrictions on data availability

Source data for uncropped gel images and data underlying reported averages are provided as a Source Data file. Additional data are available from the corresponding author upon reasonable request.

# Field-specific reporting

Please select the one below that is the best fit for your research. If you are not sure, read the appropriate sections before making your selection.

- [x] Life sciences
- [ ] Behavioural & social sciences
- [ ] Ecological, evolutionary & environmental sciences

For a reference copy of the document with all sections, see nature.com/documents/nr-reporting-summary-flat.pdf

# Life sciences study design

All studies must disclose on these points even when the disclosure is negative.

| | |
|---|---|
| Sample size | No sample-size calculations were performed. Sample size was determined to be adequate based on the magnitude and consistency of measurable differences between groups. For experiments where significance was calculated, n of at least 3 was pre-determined. |
| Data exclusions | Exclusion criteria were pre-established. In experiments with MCF-10A were excluded when cells are of suboptimal confluency and/or unhealthy (<30% of control cells in S/G2 24hrs after mitogen release or when asynchronously cycling). In small intestinal crypt experiments, crypts were randomly selected for analysis based on the following criteria: (1) contains at least ten cells expressing the sensors, (2) shaped like the letter U, and (3) contains post-mitotic cells as negative controls in the bottom of the crypt. |
| Replication | Attempts at replication were successful. Number of times experiments were repeated can be found in the figure legends. Experiments where significance were derived have at least n=3. |
| Randomization | In cell line experiments, no randomization was required because the experimented cells came from the same source and were treated identically until the variable treatment. In in vivo experiments, mice were chosen randomly to be in control or variable group. |
| Blinding | No blinding was required for the cell culture part of the study since the same automated analysis pipeline was applied to all conditions. For the in vivo analysis, the crypts were randomly selected following the criteria listed in the Data Exclusion section. The same unbiased analysis was then applied to the crypts. |

# Reporting for specific materials, systems and methods

We require information from authors about some types of materials, experimental systems and methods used in many studies. Here, indicate whether each material, system or method listed is relevant to your study. If you are not sure if a list item applies to your research, read the appropriate section before selecting a response.

## Materials & experimental systems

| n/a | Involved in the study | |
|---|---|---|
| ☐ | ☒ | Antibodies |
| ☐ | ☒ | Eukaryotic cell lines |
| ☒ | ☐ | Palaeontology and archaeology |
| ☐ | ☒ | Animals and other organisms |
| ☒ | ☐ | Human research participants |
| ☒ | ☐ | Clinical data |
| ☒ | ☐ | Dual use research of concern |

## Methods

| n/a | Involved in the study | |
|---|---|---|
| ☒ | ☐ | ChIP-seq |
| ☒ | ☐ | Flow cytometry |
| ☒ | ☐ | MRI-based neuroimaging |

## Antibodies

| | |
|---|---|
| Antibodies used | Rb(p-S807/811) (Cell Signaling, #8516), Rb (Cell Signaling, #9309), Cyclin E (Santa Cruz, sc-247), p21 (Cell Signaling, #2947), geminin (Human Atlas, HPA049977), c-Myc (Cell Signaling, #5605), γH2A.X(S139) (Cell Signaling, #2577), 53BP1 (EMD Millipore MAB3802). Rb (BD Biosciences #554136). Alexa Fluor goat secondary antibodies (ThermoFisher). Cdh1/Fzr1 (Abcam, AB89535), Emi1/Fbxo5 (Invitrogen, 37-6600), GAPDH (Cell Signaling, #5174). |
| Validation | Antibodies validated through siRNA knockdown and cellular localization using immunofluorescence: Rb(p-S807/811) (Cell Signaling, #8516), Rb (Cell Signaling, #9309), Cyclin E (Santa Cruz, sc-247), p21 (Cell Signaling, #2947), geminin (Human Atlas, HPA049977), c-Myc (Cell Signaling, #5605), Rb (BD Biosciences #554136).<br>Antibodies validated using DNA damage reagents and cellular localization using immunofluorescence: γH2A.X(S139) (Cell Signaling, #2577), 53BP1 (EMD Millipore MAB3802).<br>Antibodies validated using Western blots (band size and siRNA knockdown): Cyclin E (Santa Cruz, sc-247, 1:400), p21 (Cell Signaling, #2947, 1:1000), Cdh1/Fzr1 (Abcam, AB89535 1:1000), c-Myc (Cell Signaling, #5605, 1:1000), Emi1/Fbxo5 (Invitrogen, 37-6600, 1:500). GAPDH (Cell Signaling, #5174) validated with just band size. |

# Eukaryotic cell lines

Policy information about cell lines

| | |
|---|---|
| Cell line source(s) | MCF-10A (ATCC, #CRL-10317, human female), BJ-5ta (ATCC, #CRL-4001, human male), HS68 (ATCC, #CRL-1635, human male), WI-38 (ATCC, #CCL-75, human female), MDA-MB-435 (ATCC, #HTB-129, human female), Colo-205 (ATCC, #CCL-222, human male), U2OS (human female), acquired from the laboratory of Karlene Cimprich, HeLa (ATCC, #CCL-2, human female), Panc 08.13 (ATCC, #CRL-2551, human male), SCC-9 (ATCC, #CRL-1629, human male). |
| Authentication | MCF-10A were validated using RNA-seq. Other cell lines were not authenticated. |
| Mycoplasma contamination | MCF-10A were tested mycoplasma negative. Other cell lines were not tested. |
| Commonly misidentified lines (See ICLAC register) | MDA-MB-435 is a melanoma cell line that was misidentified as breast carcinoma in the past. The lineage of this cell line is not important for interpreting the results presented in this study. |

# Animals and other organisms

Policy information about studies involving animals; ARRIVE guidelines recommended for reporting animal research

| | |
|---|---|
| Laboratory animals | The study used Mus musculus (homozygous, constitutive expression of mCherry-hCdt1(30-120)-T2A-Venus-hGem(1-110), RDB13080, RIKEN). Both female and male mice were used, and the age ranged from 6 to 11 weeks. Mice housing conditions: temperature, 24C ±2C; humidity, 50%±10%; dark/light cycle, 12:12 hours. |
| Wild animals | The study did not involve wild animals. |
| Field-collected samples | The study did not involve samples collected from the field. |
| Ethics oversight | The animal protocols were reviewed and approved by the Animal Care and Use Committee of Kyoto University Graduate School of Medicine (No. 18086). |

Note that full information on the approval of the study protocol must also be provided in the manuscript.
